# On the Preparation of Tannic Acid

**Published:** 1853-07

**Authors:** 


*On the Preparation of Tannic Acid.*—On testing the method prescribed in the Prussian Pharmacopœia, for the preparation of tannic acid, Sandrock finds that it does not fulfil the desired object. In directing that water should be added to the ether employed, the authors of the Pharmacopœia would appear to have aimed at an approximation to the method originally adopted by Pelouze, in which crude ether was used; and to have assumed that when watery ether is used, the lower layer of the percolated liquid is a solution of tannic acid in water. However, Mohr found that this is not the case, but that the lower layer is a solution of tannic acid in ether; and Sandrock has obtained the same result on repeating his experiments. The addition of water to the ether is, therefore, useless, and moreover injurious, for the solution of tannic acid in ether is so thick that the percolation goes on very slowly, and sometimes stops altogether. The use of pure ether is open to the same objection.

The extraction of the tannic acid from galls may on the contrary be effected with ease by crude ether, and on account of the small quantity of alcohol which it contains. The alcohol facilitates the percolation by rendering the solution of tannic acid less viscid.

Instead of crude ether a mixture of sixteen parts ether and one part alcohol may be used with equally satisfactory results. The percolated liquid separates into two layers. The lower one containing the tannic acid may easily be separated, and yields a perfectly pure product on evaporation. The upper layer contains the gallic acid, coloring matter, and some tannic acid.

When a mixture of eight parts ether and one part alcohol is employed, the percolate still separates into two layers, but the lower one is smaller than when the proportion of alcohol is less, and the upper layer contains a considerably larger quantity of tannic acid.

Finally, when a mixture of four parts ether and one part alcohol is employed, the percolate does not separate into two layers, and it is difficult to separate the tannic acid from the impurities with which it is mixed.

By means of the above process a much larger product of tannic acid may be obtained than with either pure or watery ether. The tannic acid remaining in the upper layer may likewise be obtained by evaporating the liquid to dryness, treating the residue with pure ether, until the lower of the two layers into which the liquid separates no longer presents a green color. It is then separated, filtered, if necessary a little alcohol added, and evaporated.

The process recommended by Mohr, of treating the galls with a mixture of alcohol and ether in equal volumes, then evaporating the percolate which does not separate into layers, and regarding the residue as tannic acid, is altogether inadmissible, inasmuch as it gives a very impure product.—*Pharm. Journ.* from *Archiv. de Pharmacie.*

